# MTBVAC vaccination protects rhesus macaques against aerosol challenge with *M. tuberculosis* and induces immune signatures analogous to those observed in clinical studies

**DOI:** 10.1038/s41541-020-00262-8

**Published:** 2021-01-04

**Authors:** Andrew D. White, Laura Sibley, Charlotte Sarfas, Alexandra Morrison, Jennie Gullick, Simon Clark, Fergus Gleeson, Anthony McIntyre, Cecilia Lindestam Arlehamn, Alessandro Sette, Francisco J. Salguero, Emma Rayner, Esteban Rodriguez, Eugenia Puentes, Dominick Laddy, Ann Williams, Mike Dennis, Carlos Martin, Sally Sharpe

**Affiliations:** 1 Public Health England, National Infection Service, Porton Down, Salisbury, SP4 0JG UK; 2 The Churchill Hospital, Headington, Oxford, UK; 3 La Jolla Institute for Allergy and Immunology, La Jolla, CA USA; 4 Biofabri, Ponteverdra, Spain; 5 Aeras, Rockvile, MD 20850 USA; 6 Grupo de Genética de Micobacterias, Departamento Microbiología, Universidad de Zaragoza, IIS-Aragón, CIBERES, Zaragoza, Spain

**Keywords:** Tuberculosis, Live attenuated vaccines

## Abstract

A single intradermal vaccination with MTBVAC given to adult rhesus macaques was well tolerated and conferred a significant improvement in outcome following aerosol exposure to *M. tuberculosis* compared to that provided by a single BCG vaccination. Vaccination with MTBVAC resulted in a significant reduction in *M. tuberculosis* infection-induced disease pathology measured using in vivo medical imaging, in gross pathology lesion counts and pathology scores recorded at necropsy, the frequency and severity of pulmonary granulomas and the frequency of recovery of viable *M. tuberculosis* from extrapulmonary tissues following challenge. The immune profiles induced following immunisation with MTBVAC reflect those identified in human clinical trials of MTBVAC. Evaluation of MTBVAC- and TB peptide-pool-specific T-cell cytokine production revealed a predominantly Th1 response from poly- (IFN-γ^+^TNF-α^+^IL2^+^) and multi-(IFN-γ^+^TNF-α^+^) functional CD4 T cells, while only low levels of Th22, Th17 and cytokine-producing CD8 T-cell populations were detected together with low-level, but significant, increases in CFP10-specific IFN-γ secreting cells. In this report, we describe concordance between immune profiles measured in clinical trials and a macaque pre-clinical study demonstrating significantly improved outcome after *M. tuberculosis* challenge as evidence to support the continued development of MTBVAC as an effective prophylactic vaccine for TB vaccination campaigns.

## Introduction

Tuberculosis (TB) is the leading cause of death of humans from a single infectious agent worldwide. It is estimated that 10 million people fell ill with TB in 2018 and TB was responsible for 1.5 million deaths^1^. In total, 1.7 billion people are estimated to be latently infected with TB, and 10% of these individuals are at high risk of relapsing with active disease during their lifetime. The emergence of multidrug-resistant and extensively drug-resistant strains of *Mycobacterium tuberculosis* (*M. tuberculosis*), together with the geographical overlap between the HIV and TB epidemics, mean that there is an urgent need for better control of TB. Vaccination is the most effective way to control any infectious disease; however, the only vaccine currently available against TB, *Mycobacterium bovis* bacillus Calmette-Guérin (BCG), whilst effective against severe manifestations of infant tuberculosis^2^, is only partially effective against adult pulmonary TB^3^.

Non-human primate (NHP) models provide the most relevant pre-clinical models of human disease and play a critical role in vaccine development. Macaque models of *M. tuberculosis* have been established^4–7^ within which BCG has been shown to confer low-level efficacy against infectious challenge^8^, thus providing an arena in which new vaccine regimens could demonstrate superior efficacy relative to both unvaccinated or BCG-vaccinated individuals. Immune signatures that associate with an improved outcome of challenges have been identified in macaque studies^8–10^, and because of the similarities between the macaque and human immune systems and the response to TB infection^11^, it is considered that such immune signatures would be relevant to humans. Thus far it has not been possible to verify this assumption or validate putative correlates of protection because there have been insufficient parallels between TB vaccine clinical trials and pre-clinical vaccine efficacy studies showing improved outcome in the non-human primate model for comparisons to provide meaningful data.

MTBVAC is a live-attenuated strain of *M. tuberculosis* derived from a clinical isolate belonging to modern lineage 4, which is known to have a worldwide pattern of distribution. MTBVAC was designed to stimulate specific host immune responses mimicking natural TB infection without causing disease through rational attenuation by deletion of the major virulence genes *phoP* and *fadD26*^12^. MTBVAC contains all antigens present in *M. tuberculosis*, including those contained in the RD1 region, that are absent from BCG and have been associated with improved protection in animal models^13^. Since 2012, MTBVAC has been the only live-attenuated *M. tuberculosis*-based vaccine candidate in clinical trials. The safety and immunogenicity of MTBVAC were demonstrated in adults in a Phase Ia trial^14^, and in neonates by a Phase Ib trial conducted in an endemic country, which showed that MTBVAC is as safe as BCG and more immunogenic^15^. MTBVAC is now in Phase IIa dose-defining trials in both adolescents and newborns in South Africa (NCT02933281 and NCT03536117). Furthermore, data from pre-clinical testing in small animal models suggest that MTBVAC vaccination is safe, immunogenic and has the potential to enhance protection against experimental *M. tuberculosis* challenge relative to BCG^12,16,17^. Evaluation of the immunogenicity and efficacy of MTBVAC in the macaque *M. tuberculosis* challenge model would provide data to accelerate and assist product development pathway decisions to move to efficacy trials.

This study aimed to determine the protective efficacy against low-dose aerosol challenge with *M. tuberculosis* conferred by a single intradermal vaccination with MTBVAC to rhesus macaques; to characterise the immune response induced following vaccination and to compare the immune signatures defined in macaques with those defined in MTBVAC immunised humans.

## Results

### Vaccination

All animals in the study showed the weight gain profiles expected in normal healthy animals during the period prior to challenge and were unperturbed by vaccination (Supplementary Fig. 1A). Body temperature, erythrocyte sedimentation rate (ESR) and red cell haemoglobin concentration level remained within the normal range for the species during the period between vaccination and challenge in all individuals (Supplementary Fig. 1B–D). Mild induration and erythema occurred at the site of immunisation in all the animals that received an intradermal vaccination with BCG and in five of the eight macaques that received intradermal vaccination with MTBVAC. The skin reactions induced were comparable in size and resolved within six weeks after MTBVAC vaccination and between six and fourteen weeks after BCG (Supplementary Fig. 1E).

### *M. tuberculosis* exposure and challenge outcome

**Fig. 1:**
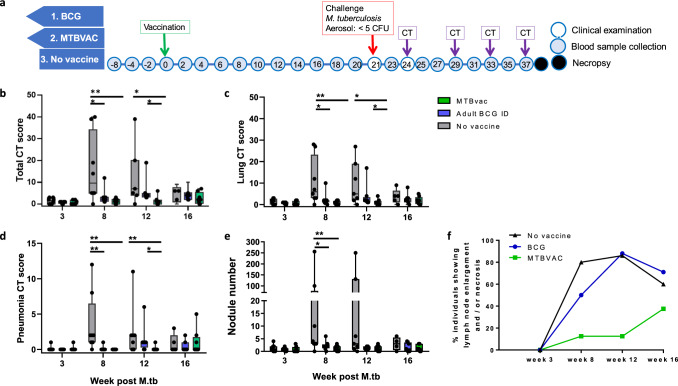
Study schedule and in vivo CT imaging. **a** Diagram showing the week in which clinical examinations (open circle), blood sample collections (shaded circle), CT scan collection, the aerosol challenge with *M. tuberculosis* and necropsy (black circle) were conducted relative to vaccination. Disease burden development quantified from CT scans reflects scores derived for total disease burden (**b**), pulmonary disease burden (**c**), pneumonia (**d**) and the number of TB-induced nodules in the lung (**e**) from CT scans collected eat weeks 3, 8, 12 and 16 after challenge with *M. tuberculosis*. Box plots show group median values +/− IQR with minimum and maximum values indicated by box whiskers. **f** Incidence of lymph node disease across the 16-week study period. Non-parametric Mann–Whitney *U* tests were used for comparison between groups with unadjusted results reported as: *P ≤ 0.05; **P ≤ 0.005.

Twenty-one weeks after vaccination, the macaques were exposed to aerosols containing an average of 27 CFU (range 14–30 CFU) *M. tuberculosis* Erdman providing an estimated median retained dose in the lung of 4 CFU. To monitor disease development, CT scans were collected 3, 8, 12 and 16 weeks after aerosol exposure and disease burden evaluated using a quantitative score system based on the extent and features of the disease visible. Whilst disease burden was comparable across groups three weeks after challenge, at week 8, significantly improved outcomes were observed in the two vaccinated groups relative to the unvaccinated group (Fig. 1a–d) (MTBVAC group: total CT score: *P* = 0.0044, lung CT score: *P* = 0.0050, number of pulmonary nodules: *P* = 0.0008, pneumonia burden: *P* = 0.0014; BCG group: total CT score: *P* = 0.0238, lung CT score: *P* = 0.0286, number of pulmonary nodules: *P* = 0.0068, pneumonia burden: *P* = 0.0039). At week 12 (Fig. 1f–i), the disease burden in the MTBVAC group remained significantly lower than in both the unvaccinated group (total CT score: *P* = 0.0208, lung CT score: *P* = 0.0249, pneumonia burden: *P* = 0.0182), and the BCG group (total CT score: *P* = 0.0073, lung CT score: *P* = 0.0407, pneumonia burden: *P* = 0.0350). A trend for reduced incidence of lymph node involvement, identified as enlargement and/or necrosis, was seen in the group that received MTBVAC (Fig. 1j) in comparison to that seen in the groups that received BCG, or remained unvaccinated.

**Fig. 2:**
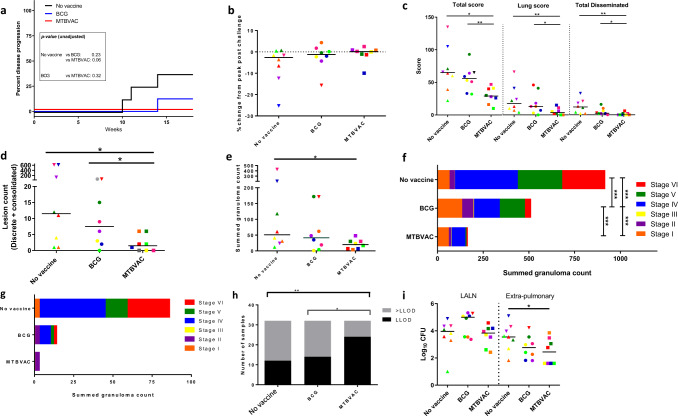
Tuberculosis-induced disease burden. **a** Kaplan–Meier plot showing the development of progressive disease to a level that met humane endpoint criteria in vaccinated and unvaccinated macaques after challenge with *M. tuberculosis*. Unadjusted *P* values from log-rank comparisons are shown. **b** Change in body weight expressed as a percentage of the peak weight measured during the post-challenge study period. **c** Total, pulmonary and disseminated (spleen, liver, kidneys) tuberculosis-induced disease burden measured using a gross pathology score system. **d** The number of macroscopic lesions in the lungs following serial sectioning. **e** Total number of granulomas (stage I–VI combined) identified in the lung from representative H&E-stained sections. **f** Total number of granulomas (stage I–VI combined) identified in the lung from representative H&E-stained sections where stacked bars indicate the number of granulomas at each stage I–VI and the combined total within each experimental group. **g** Total number of granulomas (stage I–VI combined) identified in the extrapulmonary tissues (spleen, liver, kidneys) from representative H&E-stained sections. **h** The proportion of tissue samples cultured from which *M. tuberculosis* was isolated (>LLOD: CFU value greater than the lower limit of detection of the assay; LLOD: value recorded as the lower limit of detection of the assay). **i** Bacterial burden determined in lung-associated lymph nodes (LALN) and extrapulmonary tissues. The colour and symbol coding per individual is consistent throughout and between figures. Upside down triangular symbols indicate animals in which disease progressed to meet humane endpoint criteria. Non-parametric Mann–Whitney *U* tests were used for group-wise comparisons of pathology scores, granuloma/lesion counts and viable CFU counts recovered from tissues, with unadjusted results reported as: *P ≤ 0.05; **P ≤ 0.01*. Χ*^2^ tests were used to compare the proportion of tissues with viable *M. tuberculosis* CFU counts above or below the LLOD of the assay between vaccination groups and Cochran–Armitage method *Χ*^2^ tests *to* compare granuloma stage scores between groups, unadjusted results are reported as *P ≤ 0.05; **P ≤ 0.01; ***P ≤ 0.001.

During the post-*M. tuberculosis-*exposure period, one of the BCG-vaccinated animals and three of the unvaccinated animals showed changes in behaviour and clinical parameters (weight loss, anaemia or dyspnoea) consistent with progression of the tuberculosis-induced disease that met humane endpoint criteria and was euthanized ahead of the planned end of the study. None of the animals in the MTBVAC group, or the remaining animals in the BCG and unvaccinated groups, showed adverse behavioural or premortem clinical indicators at the time of termination. The proportion of, and time at which, animals progressed to meet pre-defined humane endpoint criteria were plotted and vaccination groups compared using the log-rank test (Fig. 2a). This analysis indicated trends for improved control of disease progression during the first 16 weeks following TB infection in animals that had been vaccinated with BCG, or MTBVAC, in comparison to the unvaccinated group (MTBVAC vs unvaccinated: *P* = 0.063, BCG vs unvaccinated: *P* = 0.23).

At the end of the study, a range of approaches were applied to measure the level of tuberculosis-induced disease burden. Changes in body weight, measured at necropsy relative to the peak weight measured during the post-challenge period, were apparent in unvaccinated animals and animals that had been removed from the study due to disease progression criteria (Fig. 2b). TB-associated, pathological changes were quantified at necropsy using a gross pathology score system (Fig. 2c) and revealed that gross pathology was significantly reduced in the group that received MTBVAC compared to macaques vaccinated with BCG (total pathology score: *P* = 0.005; lung pathology score: *P* = 0.021; disseminated pathology score: *P* = 0.004), and unvaccinated macaques (total pathology score: *P* = 0.015; lung pathology score: *P* = 0.003; disseminated pathology score: *P* = 0.003).

Pulmonary disease burden measured by the manual counting of both discrete and coalesced lesions from serial sections revealed significantly fewer macroscopic lesions in the lungs from the MTBVAC-vaccinated group compared to either the BCG-vaccinated group (*P* = 0.04) or the unvaccinated group (*P* = 0.025) (Fig. 2d). Microscopic granulomas were identified in H&E-stained sections prepared from representative lung lobe and extrapulmonary tissue samples, classified into six stages and counted. Granulomas were most prevalent in the pulmonary tissues from the unvaccinated group and significantly less abundant in the group that received MTBVAC (*P* = 0.02) (Fig. 2e). Comparison of the combined number of each type of granuloma identified in the sections examined from pulmonary (Fig. 2f) and extrapulmonary (Fig. 2g) tissues revealed granulomas of all stages were observed in all groups, but the number and distribution varied amongst groups. Type V and type VI granulomas, representing more advanced lesion development, were most numerous in the unvaccinated group making up approximately half of the granulomas observed in tissue sections; by contrast, type V and type VI granulomas made up a smaller proportion of the granulomas observed in the group that received MTBVAC. The number of acid-fast bacilli (AFB) associated with each granuloma increased alongside the development of granulomas. AFB were not observed within type I or II granulomas collected from any of the macaques and only at low numbers in granulomas of type III, IV and V. The majority of AFB were found within the necrotic cores of type VI granulomas. AFB were most prevalent in granulomas from the unvaccinated group and less abundant in those from vaccinated groups, with the fewest counted in those from the MTBVAC-vaccinated group (Supplementary Table 1).

The spleen, kidneys, liver and lung-associated lymph nodes (LALN) were sampled for the presence of viable *M. tuberculosis* post-mortem. Bacteria were cultured from significantly fewer of the samples collected from the macaques given MTBVAC than from those collected from the BCG-vaccinated group (*P* = 0.011) or the unvaccinated control group (*P* = 0.003) (Fig. 2h). While the bacterial burden measured in the LALN was similar across all the study groups, the burden measured in the extrapulmonary tissues collected from MTBVAC-vaccinated (*P* = 0.0337) and the BCG-vaccinated animals was reduced compared to that measured in tissues from unvaccinated animals (Fig. 2i).

### Immune responses following vaccination and infection

**Fig. 3:**
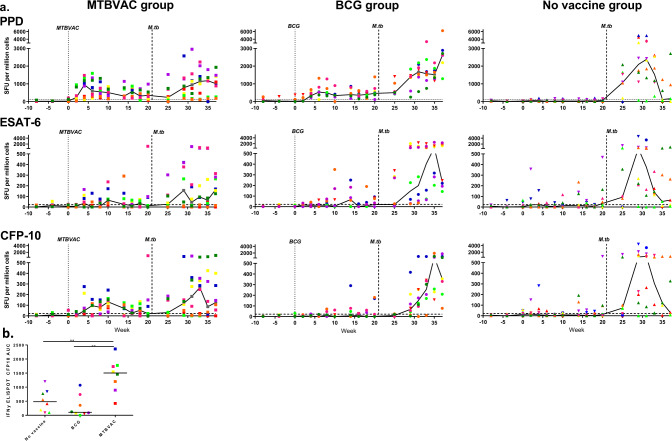
Immune response to vaccination and challenge. The frequency of *M. tuberculosis* antigen-specific IFN-γ-secreting cells induced following BCG vaccination and challenge measured by ELISpot (**a**). Top row: PPD-specific response profiles, middle row: ESAT6-specific response profiles, bottom row: CFP10-specific response profiles. Left: response profiles in the MTBVAC-vaccinated group; middle: response profiles in the BCG-vaccinated group; right: response profiles in the unvaccinated group. Vaccination with MTBVAC, or BCG indicated by the dotted line, and aerosol exposure to *M. tuberculosis* indicated by the dashed line at week 21. Comparison of the CFP10*-*specific IFN-γ response measured by ELISpot induced following vaccination with BCG or MTBVAC determined by analysis of the area under the response curve between week 2 and 20 after vaccination with group comparison made using Mann–Whitney between groups **P ≤ 0.005 (**b**). All line graphs show individual response (circular symbol) and Group median response (solid line). The colour and symbol coding per individual is consistent throughout and between figures. Upside down triangular symbols indicate animals in which disease progressed to meet humane endpoint criteria. The assay threshold (mean of pre-vaccination values plus 1.5× standard deviation) is indicated by a horizontal dotted line (PPD: 119 spot forming units (SFU) per million cells; ESAT6: 40 SFU per million cells; CFP10: 28 SFU per million cells).

The mycobacterium-specific IFN-γ response induced by vaccination and *M. tuberculosis* challenge was measured using an ex vivo ELISpot assay applied at 2-week intervals through the study (Fig. 3a). In the period before the challenge, PPD-specific IFN-γ spot forming unit (SFU) frequencies significantly increased above the threshold in all macaques that received vaccinations with BCG or MTBVAC (BCG: *P* = 0.0002; MTBVAC: *P* = 0.0002). The peak PPD-specific response occurred four weeks after vaccination in the MTBVAC group and 2 weeks later in the BCG-vaccinated group at week 6. Twenty weeks after vaccination and 1 week before challenge with *M. tuberculosis*, PPD-specific IFN-γ SFU frequencies measured in the vaccinated groups were significantly higher than the levels measured in the unvaccinated group (BCG: *P* = 0.0006; MTBVAC: *P* = 0.0019). CFP10-specific IFN-γ SFU frequencies exceeding the assay threshold between vaccination and challenge were only detected in the MTBVAC group and consequently, the response was significantly higher than those in the BCG-vaccinated (*P* = 0.0011) and the unvaccinated groups (*P* = 0.0033), with the largest responses seen between 6 and 10 weeks (Fig. 3b). During the same period between vaccination and challenge, ESAT6-specific SFU frequencies did not increase above background levels in any of the test groups. Following exposure to *M. tuberculosis*, reduced frequencies of PPD, CFP10 and ESAT6-specific SFU were seen in the MTBVAC and BCG-vaccinated groups relative to the unvaccinated group, with the differences reaching significance for PPD and ESAT6 in the MTBVAC-vaccinated group (PPD: *P* = 0.0030; ESAT6: *P* = 0.0030) and for PPD and CFP10 in the BCG-vaccinated group (PPD: *P* = 0.0104; CFP10: *P* = 0.0070). Fourteen weeks after the challenge, the MTBVAC group showed significantly lower levels of PPD-, CFP10- and ESAT6-specific SFU in the periphery than those recorded in the BCG-vaccinated group (PPD*: P* = 0.0379; CFP10*: P* = 0.0148; ESAT6: *P* = 0.0148) which could be interpreted as better control of the *M. tuberculosis* infection.

**Fig. 4:**
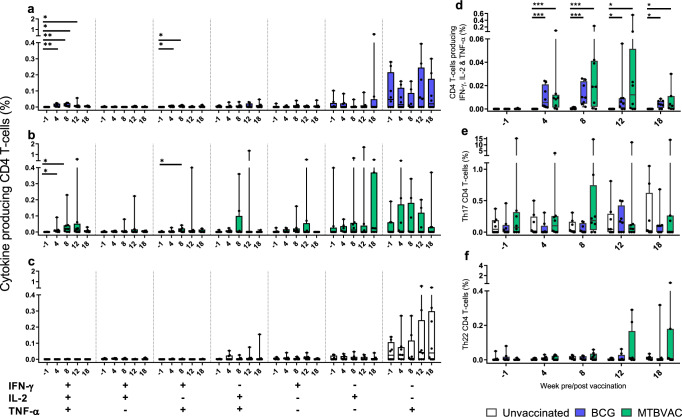
*M. tuberculosis* peptide-specific CD4 T-cell cytokine secretion profiles measured by whole blood intracellular cytokine staining. Box plots show the vaccination group median frequency of CD4 T-cells producing combinations of the cytokines IFN-γ, IL-2 and TNF-α (**a**–**d**); IL-17 (**e**) or IL-22 (**f**) +/− IQR with minimum and maximum values indicated by box whiskers. Cytokine production was measured at weeks prior to (−1), and following (4, 8, 12 and 18) BCG or MTBVAC vaccination. Significant differences between pre- and post-vaccination values (Wilcoxon signed-rank) and between groups (Mann–Whitney *U* test) are indicated by bars and asterisks: *P ≤ 0.05, **P ≤ 0.01, ***P ≤ 0.001 (all *P* values are unadjusted for multiple comparisons). Frequencies measured in individual animals are represented by dots.

Multiparameter flow cytometry assays were applied to explore the phenotype and functional profile of the cellular immune response. Application of intracellular cytokine staining to measure antigen-specific cytokine production revealed increased frequencies of multifunctional CD4 T-cells producing IFN-γ, IL-2 and TNF-α (polyfunctional), or IFN-γ and TNF-α, in animals that received BCG or MTBVAC, with the frequency of cytokine-producing cells reaching significance above pre-vaccination levels from four weeks after vaccination (Fig. 4a, b and Supplementary Fig. 2A, B). Cytokine-producing CD4 T-cell populations remained unchanged in the unvaccinated group consistent with the lack of experimental intervention applied to this group at this stage of the experiment (Fig. 4c and Supplementary Fig. 2C). Comparison of multifunctional T-cell frequencies between the vaccination groups indicated that significantly higher frequencies of CD4 T-cells producing IFN-γ, IL-2 and TNF-α (Fig. 4d); or IFN-γ and TNF-α (comparative plot not shown) simultaneously were present in the BCG- and MTBVAC-vaccinated groups relative to the unvaccinated animals at weeks 4, 8, 12 and 18. Changes in the frequency of antigen-specific CD4 T-cells producing IL-17 or IL-22 were not detected following vaccination and differences were not apparent between the vaccination groups (Fig. 4e, f), indicating that both BCG and MTBVAC vaccination induced a predominantly Th1 CD4 T-cell response. Vaccination-induced cytokine-producing CD8 T-cell populations were detected at a low frequency relative to the CD4 T-cell subsets and generally did not increase significantly above pre-vaccination levels (Supplementary Figs. 3 and 4). Similarly, potential vaccination-induced changes in the frequency of innate lymphoid cells were explored by quantification of mucosal-associated invariant T cells (MAIT’s) and natural killer (NK) cell subsets. However, MAIT cell frequencies detected in BCG, MTBVAC and unvaccinated groups remained at low frequency (group medians below 1% of the total T-cell population) following vaccination (Supplementary Fig. 5A), and NK cell populations (identified by the pattern of CD56 and CD16 expression) did not differ significantly at any time point in comparison to pre-vaccination levels or between the vaccination groups (Supplementary Fig. 5B). Furthermore, cytokine production from the NK cell population as a whole (regardless of CD56 and CD16 expression) was assessed (Supplementary Fig. 5C–H), but significant differences were not detected in the total frequency of NK cells producing IFN-γ, IL-2, TNF-α, IL-17 or IL-22 following MTBVAC or BCG vaccination in comparison to pre-vaccination levels.

Antigen-specific cytokine production by γδ T-cell populations was found to primarily consist of TNF-α and IL-17 producing cells, with higher frequencies detected in response to stimulation with MTBVAC rather than pooled *M. tuberculosis*-derived peptides (Supplementary Figs 6 and 7). Trends for a vaccine-induced increase in the frequency of cytokine-producing γδ T-cell populations were apparent in both the BCG and MTBVAC-vaccinated groups; however, these responses failed to reach significance above pre-vaccination levels (Supplementary Figs 6 and 7). Comparison of IL-17-producing γδ T-cell frequencies between groups at each time point following vaccination (Supplementary Fig. 6) indicated a trend for increased production of IL-17 at weeks 4 and 8 following MTBVAC vaccination, whereas by weeks 12 and 18 greater frequencies were measured in the BCG-vaccinated group. Despite these trends, the differences measured between the vaccination groups did not reach statistical significance. Nevertheless, as a large proportion of the γδ T-cell population produced antigen-specific IL-17 following vaccination, which was in contrast to the low frequency of IL-17-producing CD4 and CD8 T cells, this indicates a potentially important role for the γδ T-cell subset in the production of this important immunomodulatory cytokine.

## Discussion

Intradermal vaccination with MTBVAC was well tolerated by adult rhesus macaques and conferred a significant improvement in outcome following aerosol exposure to *M. tuberculosis* compared to that provided by BCG vaccination. Statistically significant differences in disease burden development were demonstrated between groups measured using a scoring system applied to CT scan images. This approach revealed the disease burden in the MTBVAC group to be significantly reduced compared to that in the unvaccinated control group 8 weeks after challenge and significantly reduced in comparison to the BCG-vaccinated group by week 12. This finding was reflected in the results of the analyses applied at the end of the study schedule, 16 weeks after challenge, when measures based on the assessment of macroscopic and microscopic pathology and bacterial burden verified an improved outcome after challenge in the MTBVAC-vaccinated group relative to the BCG-vaccinated and the unvaccinated groups. Furthermore, after the challenge, higher levels of CFP10 and ESAT6-specific IFN-γ-secreting cells were detected in the unvaccinated relative to the vaccinated groups with the lowest levels present in the MTBVAC group. The association between increased frequencies of CFP10 and ESAT6-specific responses post-challenge with the poorer post-challenge outcome is consistent with other reports and suggests that the frequency of responses to these diagnostic antigens provides a biomarker reflective of disease progression and antigen load. The rhesus macaque provides a stringent model, in which only a few vaccines have conferred significant improvements in outcome after *M. tuberculosis* challenge^8–10,18–20^ and only one other new TB vaccine has afforded significantly superior protection compared to intradermally delivered BCG^19^. Taken together, these data support the continued development of MTBVAC as a more effective prophylactic vaccine for TB vaccination campaigns.

The immune profiles induced following immunisation with MTBVAC reflect those identified in human clinical trials^14,15,17^. Evaluation of MTBVAC- and TB peptide-pool-specific T-cell cytokine production revealed a predominantly Th1 response profile from poly- (IFN-γ^+^TNF-α^+^IL2^+^) and multi- (IFN-γ^+^TNF-α^+^) functional CD4 T cells, while only low levels of Th22, Th17 and cytokine-producing CD8 T-cell populations were detected. As in clinical trials, these profiles were similar in profile and scale to those identified after BCG vaccination; although differences in antigen recognition were detected that reflect protective immunological signatures reported from small animal studies^17^, which included the detection of low-level CFP10-specific IFN-γ responses following MTBVAC, but not BCG, vaccination. This is analogous to the findings of Aguilo et al.^17^, who have demonstrated a link between MTBVAC-induced CFP10 and ESAT6-specific IFN-γ secretion and improved protection against *M. tuberculosis* challenge in the C3H/HeNRj mouse strain, an improvement that was negated when mice were vaccinated with the ESAT6 and CFP10 ablated MTBVAC-ΔE6C10 construct. Low-level, but significant, increases in CFP10-specific IFN-γ-secreting cells have also been detected in MTBVAC-vaccinated human volunteers; although, in contrast to murine studies, ESAT6-specific cytokine production did not increase significantly in this population^14,17^. This profile of antigen specificity was mirrored directly by the cellular immune responses measured in macaques where significant increases in CFP10-, but not ESAT6-, specific IFN-γ secreting cells were detected following MTBVAC vaccination. This not only provides further evidence that a low-level CFP10-specific immune response is a key feature of the MTBVAC-induced cell-mediated response but is also a validation that the immunogenicity profiles measured in macaques are representative of immune signatures observed in humans. This concordance between immune profiles measured in clinical trials and a macaque pre-clinical assessment of a novel TB vaccine candidate demonstrating significantly improved outcome after *M. tuberculosis* challenge is a promising indication that the improved protection provided by MTBVAC vaccination to macaques and mice will translate to the human population.

## Methods

### Experimental animals

Twenty-four male and female rhesus macaques (*Macaca mulatta*) of Indian origin aged between 4.2 and 5.2 years of age were sourced from an established, characterised, closed UK breeding colony. Compatible social groups were housed in accordance with Home Office (UK)^21^ and NC3Rs guidelines^22^ in cages with high-level observation balconies, extensive environmental stimulation and provided with a wide range of dietary enrichment^23^. Animal procedures and study designs were approved by the Establishment of Animal Welfare and Ethical Review Committee and authorised under a UK Home Office project licence. Prior to challenge with *M. tuberculosis* macaques were housed in cages approximately 2.5-m high by 4-m long by 2-m deep, constructed with high-level observation balconies and with a floor of deep litter to allow foraging. Following the challenge, animals were transferred to banks of cages placed in directional airflow containment systems that allowed group housing and environmental control whilst providing a continuous, standardised inward flow of fully conditioned fresh air identical for all groups. Additional environmental enrichment was afforded by the provision of toys, swings, feeding puzzles and DVDs for visual stimulation. In addition to standard old-world primate pellets, the diet was supplemented with a selection of fresh vegetables and fruit. For each procedure, sedation was applied by intramuscular injection with ketamine hydrochloride (10 mg/kg) (Ketaset, Fort Dodge Animal Health Ltd, Southampton, UK). None of the animals had been used previously for experimental procedures, and each socially compatible group was randomly assigned to study treatment. Prior to study enrolment, previous exposure to mycobacterial antigens was assessed using an IFN-γ ELISPOT (MabTech, Nacka. Sweden) to detect responses to tuberculin-PPD (SSI, Copenhagen, Denmark), and pooled 15-mer peptides of ESAT6 and CFP10 (Peptide Protein Research LTD, Fareham, U.K.).

### Vaccination

The vaccination schedule relative to the aerosol challenge is shown in Fig. 1a. The sample size for the experiment was determined using total pathology score as the primary efficacy readout and was powered to detect a nine-point reduction (arbitrary units) with a power of 80% and an α of 0.05. Eight of the macaques were immunised by intradermal (ID) injection in the upper left arm with 100 μl of BCG vaccine, Danish strain 1331 (1st WHO reference reagent, NIBSC, UK), the second group of eight macaques received an ID vaccination with 100 µl of MTBVAC (Biofabri, Spain) and the remaining eight animals were left untreated as a negative control group. The BCG and MTBVAC vaccines were prepared for intradermal administration according to the manufacturer’s instructions for administration to humans. One ml of Sautons diluent was added to a vial of BCG vaccine to give a suspension of BCG at an estimated concentration of between 2 and 8 × 10^6^ CFU/ml and 1 ml of sterile water was added to MTBVAC to give a concentration of 3–17 × 10^6^ CFU. Due to differences in vaccine diluent solutions, it was not possible to deliver a mock vaccination to animals in the unvaccinated control group. Vaccinations were administered within 1 h of vaccine reconstitution. Plate culture of residual vaccine confirmed the viability of the BCG and MTBVAC vaccines, and the mean average administered dose of each vaccine was calculated to be 1.2 × 10^6^ (SD 3.4 × 10^4^) CFU and 8.2 × 10^5^ (SD 3.9 × 10^4^) CFU for BCG and MTBVAC respectively. The injection sites were monitored for local reactions after vaccination with BCG or MTBVAC such that reactions were measured and assessed.

### *M. tuberculosis* challenge strain

The Erdman K01 stock (HPA-Sept 2011) used for the challenge was prepared from stocks of the *M. tuberculosis* Erdman strain K01 (BEI Resources). A stock suspension was initially prepared from a 5-ml bacterial starter culture originally generated from colonies grown on Middlebrook 7H11 supplemented with oleic acid, albumin, dextrose and catalase (OADC) selective agar (BioMerieux, UK). A liquid batch culture was then grown to logarithmic growth phase in 7H9 medium (Sigma-Aldrich, UK) supplemented with 0.05% (v/v) Tween 80 (Sigma-Aldrich, UK). Aliquots were stored at −80 °C. The concentration of colony-forming units (CFU)/ml in the stock suspension was determined from thawed aliquots by enumeration of CFU cultured on Middlebrook 7H11 OADC selective agar.

### Aerosol exposure

Twenty-one weeks after vaccination, all animals were challenged by exposure to aerosols of *M. tuberculosis*, as previously described^24,25^. Mono-dispersed bacteria in particles were generated using a three-jet Collison nebuliser (BGI) and, in conjunction with a modified Henderson apparatus^26^, delivered to the nares of each sedated primate via a modified veterinary anaesthetic mask. Challenge was performed on sedated animals placed within a ‘head-out’, plethysmography chamber (Buxco, Wilmington, North Carolina, USA) to enable the aerosol to be delivered simultaneously with the measurement of respiration rate and respired volume. The calculations to derive the presented dose (PD) (the number of organisms that the animals inhale) and the retained dose (the number of organisms assumed to be retained in the lung) have been described previously^27,28^. A nebuliser concentration was selected to result in a retained dose of approximately five viable CFU.

### Clinical procedures

Full clinical examinations were conducted at 2-week intervals throughout the study. Macaques were sedated, thoracic radiographs were taken, body weight and temperature measured and blood samples collected. Red blood cell (RBC) haemoglobin levels were measured using a HaemaCue haemoglobinometer (Haemacue Ltd, Dronfield, UK), and ESR was determined using the Sediplast system (Guest Medical, Edenbridge, UK). Observed behaviour was monitored for contra-indicators. The time of necropsy, if prior to the end of the planned study period, was determined by experienced primatology staff and based on a combination of the following adverse indicators: depression or withdrawn behaviour, abnormal respiration (dyspnoea), loss of 20% of peak post-challenge body weight, ESR levels elevated above normal (>20 mm), haemoglobin level below normal limits (<100 g/dL), increased temperature (>41 °C) and abnormal findings on the thoracic radiographs.

### Computed tomography (CT) imaging

CT scans were collected from sedated animals using a 16-slice Lightspeed CT scanner (General Electric Healthcare, Milwaukee, WI, USA) 3, 8, 12 and 16 weeks after aerosol exposure to *M. tuberculosis*, as described previously^24^. To facilitate full examination of lesions and lymph nodes, Niopam 300 (Bracco, Milan, Italy), a non-ionic, iodinated contrast medium, was administered intravenously (IV) at 2 ml/kg body weight. Scans were evaluated by an expert thoracic radiologist blinded to the animal’s treatment and clinical status, for the number and distribution across lung lobes of pulmonary lesions and the presence of disease features, such as nodular conglomeration, cavitation, consolidation (an indicator of alveolar pneumonia), a ‘tree-in–bud’ pattern (an indicator of bronchocentric pneumonia) and lobular collapse. The airways were evaluated for the occurrence of wall thickening and the presence of bronchocele. The lymph nodes were assessed for enlargement and the presence of necrosis. Extrapulmonary tissues, including liver, kidneys and spleen were examined for the presence of single or multiple foci of disease, cavitation or necrosis. The disease burden attributable to infection with *M. tuberculosis* was scored using a relative scoring system based on the number of lesions present in lungs, spleen, liver, kidney and lymph nodes, and the presence and extent of TB-induced structural abnormalities as described previously^23^. The scores for consolidation and tree-in-bud were summed to provide a pneumonia score. The scores attributed to each tissue (lung lobe, organ, lymph node) were summed to give the total CT score to provide a measure of pulmonary and extrapulmonary disease burden.

### Interferon-gamma (IFN-γ) ELISpot

Peripheral blood mononuclear cells (PBMC) were isolated from heparin anti-coagulated blood using standard methods. An IFN-γ ELISpot assay was used to quantify the number mycobacteria-specific IFN-γ-producing T cells in PBMCs using a human/simian IFN-γ kit (MabTech, Nacka. Sweden), as described previously^24^. In brief, 2 × 10^5^ PBMCs were cultured with 10 μg/ml PPD (SSI, Copenhagen, Denmark) or pools of overlapping 15-mer peptides spanning CFP10 or ESAT6 (Peptide Protein Research Ltd, Wickham, UK) in duplicate, or without antigen, in quadruplicate, and incubated for 18 h. Phorbol 12-myristate (Sigma-Aldrich Dorset, UK) (100 ng/ml) and ionomycin (CN Biosciences, Nottingham, UK) (1 μg/ml) were used as a positive control. After culture, spots were developed according to the manufacturer’s instructions. Plates were scanned, and spots enumerated using a CTL Immunospot S6 reader and software. Determinations from replicate tests were averaged, and the data were analysed by subtracting the mean number of spots in the medium-only control wells from the mean counts of spots in wells with antigen, or peptide pools, to derive an antigen-specific spot count. This value was multiplied by a factor of five and reported as IFN-γ SFU frequency per million PBMCs. A minimum threshold level of activity was calculated for each mycobacterial antigen by analysis of the distribution of SFU frequencies quantified prior to mycobacterial vaccination or challenge (baseline SFU). Threshold values of 119 SFU (mean + 1.5 SD) for PPD-; 28 SFU (mean + 2.0 SD) for CFP10-; and 40 SFU (mean + 2.0 SD) for ESAT6-specific SFU were selected to encompass the 95th percentile of SFU frequencies measured at baseline. Only responses exceeding these thresholds were considered as positive. Antigen-specific IFN-γ SFU profiles were plotted using Graphpad v7.0 (Graphpad Inc, USA) and used to calculate area under the curve (AUC) values for comparison of vaccination group median AUC by Mann–Whitney *U* test.

### Whole blood intracellular cytokine staining (WB ICS) assay

#### In vitro stimulation

The method used to stimulate blood cells for ICS was closely aligned with protocols applied in MTBVAC clinical trials conducted by the South African Tuberculosis Vaccine Initiative (SATVI)^15^. In brief, WB ICS was performed using 450 µl of sodium heparin (Sigma-Aldrich, UK) anti-coagulated blood incubated for a total of 12 h with 0.25 µg/ml of anti-CD28 and anti-CD49d co-stimulatory antibodies (both from BD Biosciences, UK) and 1 µg/ml of selected *M. tuberculosis* peptides (TB Mega-pool provided by C. Lindestam-Arlehamn, La Jolla Institute for Allergy and Immunology, USA), 1 × 10^6^ CFU/ml of MTBVAC (Biofabri, Spain), 5 µg/ml of staphylococcus enterotoxin B (SEB) as a positive control (Sigma-Aldrich, UK), or sterile phosphate buffer saline (PBS) as a negative control (Severn Biotech, UK). Antigenic stimulation was initiated within 2 h of blood sample collection and proceeded for 7 h in a 37 °C water bath before 50 µl of plasma was removed from each sample and frozen at −80 °C as a resource for future interrogation of secreted biomarkers. The protein transport inhibitor Brefeldin-A (Sigma-Aldrich, UK) was added to the mixture at a final concentration of 10 µg/ml, and the samples returned to incubation conditions for a further 5 h. Following the completion of the incubation period, EDTA solution (Sigma-Aldrich, UK) was added at a concentration of 2 mM to detach adherent cells. After 15 min, the sample was diluted 1:10 in FACSlyse solution (BD Biosciences, UK) and incubated for 15 min to remove red blood cell contamination. Finally, the cellular fraction of the sample was separated by centrifugation and resuspended in 1 ml of cryosolution consisting of 30% foetal calf serum (Labtech,UK), 20% DMSO and 50% RPMI culture medium (both from Sigma-Aldrich, UK), before freezing under a controlled cooling rate and cryopreservation in liquid nitrogen vapour phase.

Samples were thawed in batches for flow cytometry analysis such that all the samples from an individual animal were stained with antibodies and analysed on the same occasion. The cryopreserved material was rapidly thawed at 37 °C and washed twice by tenfold dilution in RPMI + 1U/ml DNase (Sigma-Aldrich, UK) followed by centrifugation. Cells were permeabilised using BD cytofix/cytoperm reagent, washed and resuspended in 50 µl perm wash buffer (BD Biosciences, UK) for surface and intracellular antibody staining. The cell surface and intracellular staining mixture were prepared in Brilliant Staining buffer (BD Biosciences, UK) and consisted of the following fluorochrome-conjugated antibodies: anti-CD3 AF700; anti-CD4 PerCP-Cy5.5; anti-CD161 Pe-Cy7; anti-IFN-γ FITC; anti-TNF-α BUV395 (all from BD Biosciences, UK); anti-CD8 APC-Fire750; anti-Vα7.2 PE-Dazzle 594; anti-CD16 BV785; anti-γδ-TCR BV421; anti-IL-17 BV711; anti-CD56 BV605 (all from Biolegend, UK); anti-IL-22 APC (Thermo Fisher Scientific, UK) and anti-IL-2 PE (Miltenyi Biotech, UK). BD Compbeads (BD Biosciences, UK) were labelled with the above fluorochromes for use as compensation controls. Following antibody labelling, cells and beads were washed by centrifugation and fixed in 4% paraformaldehyde solution (Sigma-Aldrich, Gillingham, UK) prior to flow cytometric acquisition.

#### Flow cytometric acquisition and analysis

Flow cytometry data was acquired using a five-laser LSRII Fortessa instrument (BD Biosciences, Oxford, UK) and data were analysed using FlowJo (version 10.5.3 BD Biosciences, UK) (Supplementary Fig. 8). Cell aggregates and debris were excluded from the analysis using forward scatter-height (FSC-H) versus forward scatter-area (FSC-A), followed by side scatter-height (SSC-H) versus side scatter-area (SSC-A) plots. Cytokine-producing lymphocyte populations were then identified using a FSC-H versus SSC-A dot plot, followed by sequential gating through CD3^+^, followed by CD4^+^, CD8^+^ or γδ-TCR^+^ gates for identification of the major T-cell populations, or CD3^−^, CD8^+^, CD56^+^ and/or CD16^+^ NK cells, before individual cytokine gates to identify IFN-γ, IL-2, IL-17, IL-22 and TNF-α producing populations. Polyfunctional T cells were identified using Boolean gating combinations of individual cytokine-producing CD4, CD8 or γδ T cells. The software package PESTLE (version 1.8) was used for background subtraction to obtain antigen-specific cytokine responses, and Graphpad (version 8.0.1) was used to generate graphical representations of flow cytometry data (Graphpad Software Inc, USA).

### Necropsy

The necropsies were conducted either when the disease progressed to meet the criteria set as a humane endpoint or 16 to 18 weeks after *M. tuberculosis* aerosol challenge. Animals were anaesthetised, and clinical data collected. Blood samples were taken prior to euthanasia by intracardiac injection of a lethal dose of anaesthetic (Dolelethal, Vétoquinol UK Ltd, 140 mg/kg). A post-mortem examination was performed immediately and gross pathological changes were scored using an established system based on the number and extent of lesions present in the lungs, spleen, liver, kidney and lymph nodes, as described previously^29^. Samples of spleen, liver, kidneys and tracheobronchial, inguinal and axillary lymph nodes were removed and sampled for quantitative bacteriology. The lung, together with the heart and attached tracheobronchial and associated lymph nodes, were removed intact. The lymph nodes were measured and examined for lesions. These were fixed by intra-tracheal infusion with 10% neutral buffered formalin (NBF) using a syringe and 13CH Nelaton catheter (J.A.K. Marketing, York, UK). The catheter tip was inserted into each bronchus in turn via the trachea; the lungs were infused until they were expanded to a size considered to be normal inspiratory dimensions, and the trachea ligated to retain the fluid. The infused lung was immersed in 10% NBF. In addition, samples of kidneys, liver, spleen, and sub clavicular, hepatic, inguinal and axillary lymph nodes were fixed in 10% NBF.

### Pathology studies

#### Gross examination following fixation

The lung lobes were sliced serially (~5-mm intervals). Discrete and coalesced lesions were counted and recorded, and the dimensions of the latter were measured. A tissue slice containing obvious lesions (when present) was chosen from each lung lobe. Where gross lesions were not visible, a sample was taken from a pre-defined anatomical location from each lobe to establish consistency between animals. Sections of LALN (those associated with the distal trachea and bifurcation) and from extra-thoracic organs described previously, were also fixed in formalin.

#### Histopathological examination

All samples were processed to paraffin wax blocks using standard procedures, sectioned at ~4 µm, stained with haematoxylin and eosin (H&E) and Ziehl-Neelsen (ZN) stains. Slides were scanned digitally using ‘3D Histech’ slide scanner and proprietary ‘Caseviewer’ software used to capture, store and annotate digital images. All gross and histopathological examinations were carried out by a qualified veterinary pathologist blinded to the treatment group. Each slide was evaluated for the presence of tuberculous lesions and AFBs. The TB-associated lesions were recorded using the scoring system described by Rayner et al.^30^. Briefly, six different types of granulomas were identified. Types one (I), two (II) and three (III) were considered as “unorganised” lesions, while types four (IV), five (V) and six (VI) were described as “organised” granulomas. Type I was small, diffuse foci of macrophages and lymphocytes with scattered neutrophils and eosinophils, lacking clearly defined margins, infiltrate alveolar walls and extend into alveoli. Type II lesions were composed of similar cell types as type I but were larger in size and forming a more defined circumscribed focus of granulomatous inflammation, frequently circular and with variably demarcated border. Type III was as type II but with focal necrosis present, characterised by nuclear pyknosis and karyorrhexis with the loss of cellular architecture. Type IV was characteristically circumscribed granulomas consisting primarily of macrophages admixed with neutrophils and other leucocytes, with evidence of a few peripheral lymphocytes. Type V was organised lesions exhibiting necrotic foci with degenerated neutrophils and type VI were classic, largely well-demarcated, granulomas with central caseous necrosis and a variable rim of lymphocytes. For each tissue section, the total area of gross disease was calculated and the total number of granulomas of each type were counted and recorded. Moreover, the total number of AFBs were counted for each granuloma in the ZN-stained sections from the lung and LALN.

### Bacteriology

The spleen, kidneys, liver and tracheobronchial lymph nodes were sampled for the presence of viable *M. tuberculosis* post-mortem, as described previously^20^. Weighed tissue samples were homogenised in 2 ml of sterile water, then either serially diluted in sterile water prior to being plated, or plated directly onto Middlebrook 7H11 OADC selective agar. Plates were incubated for three weeks at 37 °C and resultant *M. tuberculosis* colonies counted. Mean CFU counts from replicate tissue samples were used for graphical presentation. The lower limit of detection for culture on plates was five CFUs per mL of homogenate.

### Statistical analyses

GraphPad Prism version 7.0 (GraphPad Software Inc, La Jolla, California, USA) was used for all graphical data representations and statistical analyses, these included: comparison of antigen-specific IFN-γ SFU frequencies measured using the ex vivo ELISpot assay, frequencies of specific cell subsets and multifunctional cell populations measured by flow cytometry, differences in qualitative pathology scores and histopathology granuloma severity scores, quantification of viable *M. tuberculosis* in extrapulmonary tissues and comparisons of clinical parameters between vaccination groups, using non-parametric Wilcoxon signed-rank or Mann–Whitney *U* tests. Chi-squared (*Χ*^2^ tests were used to compare differences between the vaccination groups in the proportion of bacteriology tissue samples where viable *M. tuberculosis* CFU levels were below the lower limit of detection of the assay and Cochran–Armitage method *Χ*^2^ tests for the comparison of the frequency of granuloma severity data. Disease progression between groups was compared using log-rank survival analysis. Where applicable, all statistical analyses were conducted using two-tailed tests and *P* values are unadjusted for multiple comparisons.

### Reporting summary

Further information on research design is available in the Nature Research Reporting Summary linked to this article.

## Supplementary information

Supplementary Information

Reporting Summary Checklist

## Data Availability

The datasets generated and analysed in this study are available from the corresponding authors upon reasonable request. Likewise, biomaterials archived from this study may be shared for further research.
