# Supplementary material for: MTBVAC vaccination protects rhesus macaques against aerosol challenge with M. tuberculosis and induces immune signatures analogous to those observed in clinical studies
Source: NPJ Vaccines. 2021 Jan 4;6:4. doi: 10.1038/s41541-020-00262-8 (PMC7782851; doi:10.1038/s41541-020-00262-8)
Supplement: Supplementary file 1 — Supplementary Information [file 41541_2020_262_MOESM1_ESM.pdf]

Supplementary Table 1

Average number of acid fast bacilli per granuloma of each developmental stage in lung and hilar lymph node counted in Ziehl-Neelsen stained tissue sections.

Group	Mean number of acid fast bacilli per granuloma					
	Granuloma stage					
	Type I	Type II	Type III	Type IV	Type V	Type VI
BCG	0	0	0	0	0.106	8.167
MTBVAC	0	0	0	0	0	2.214
No vaccine	0	0	0	0.009	0.400	16.577

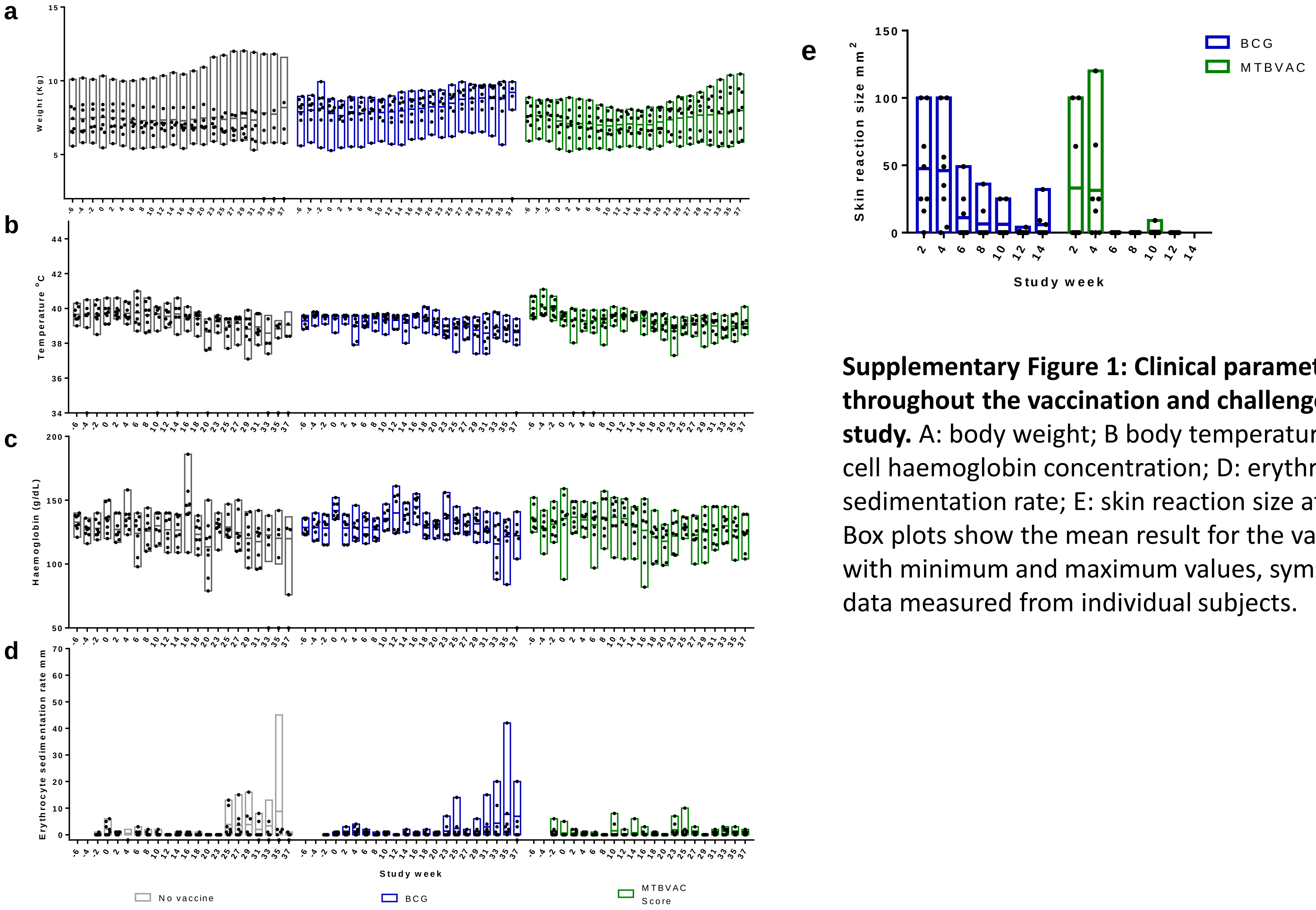

Supplementary Figure 1: Clinical parameters measured throughout the vaccination and challenge phases of the study. A: body weight; B body temperature; C: red blood cell haemoglobin concentration; D: erythrocyte sedimentation rate; E: skin reaction size at vaccination site. Box plots show the mean result for the vaccination group with minimum and maximum values, symbols represent data measured from individual subjects.

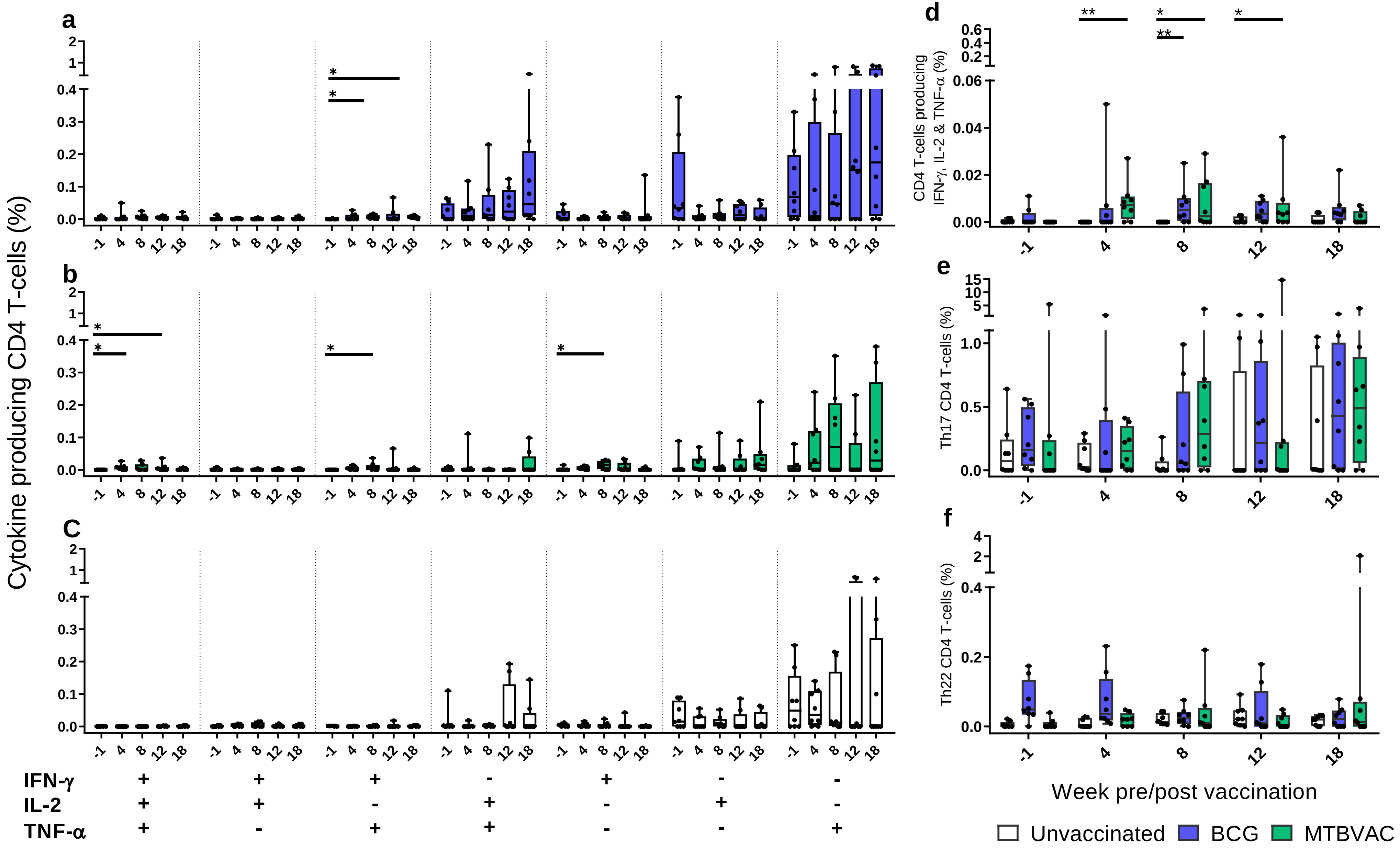

a
b
C
d
e
f
Cytokine producing CD4 T-cells (%)
CD4 T-cells producing IFN-γ, IL-2 & TNF-α (%)
Th17 CD4 T-cells (%)
Th22 CD4 T-cells (%)
IFN-γ + + + - + - -
IL-2 + + - + - + -
TNF-α + - + + - - +
Week pre/post vaccination
Unvaccinated
BCG
MTBVAC

Supplementary Figure 2. MTBVAC-specific CD4 T-cell cytokine secretion profiles measured by whole blood intracellular cytokine staining. Box plots show the vaccination group median frequency of CD4 T-cells producing combinations of the cytokines IFN-γ, IL-2 and TNF-α (A, B, C, D); IL-17 (E) or IL-22 (F) +/- IQR with minimum and maximum values indicated by box whiskers. Cytokine production was measured at specific weeks prior to (-1) and following (4, 8, 12 and 18) BCG or MTBVAC vaccination. Significant differences between pre- and post-vaccination values (Wilcoxon signed-rank) and between groups (Mann-Whitney U-test) are indicated by bars and asterisks: * $p \leq 0.05$, ** $p \leq 0.01$, *** $p \leq 0.001$ (all p-values are unadjusted for multiple comparisons). Frequencies measured in individual animals are represented by dots.

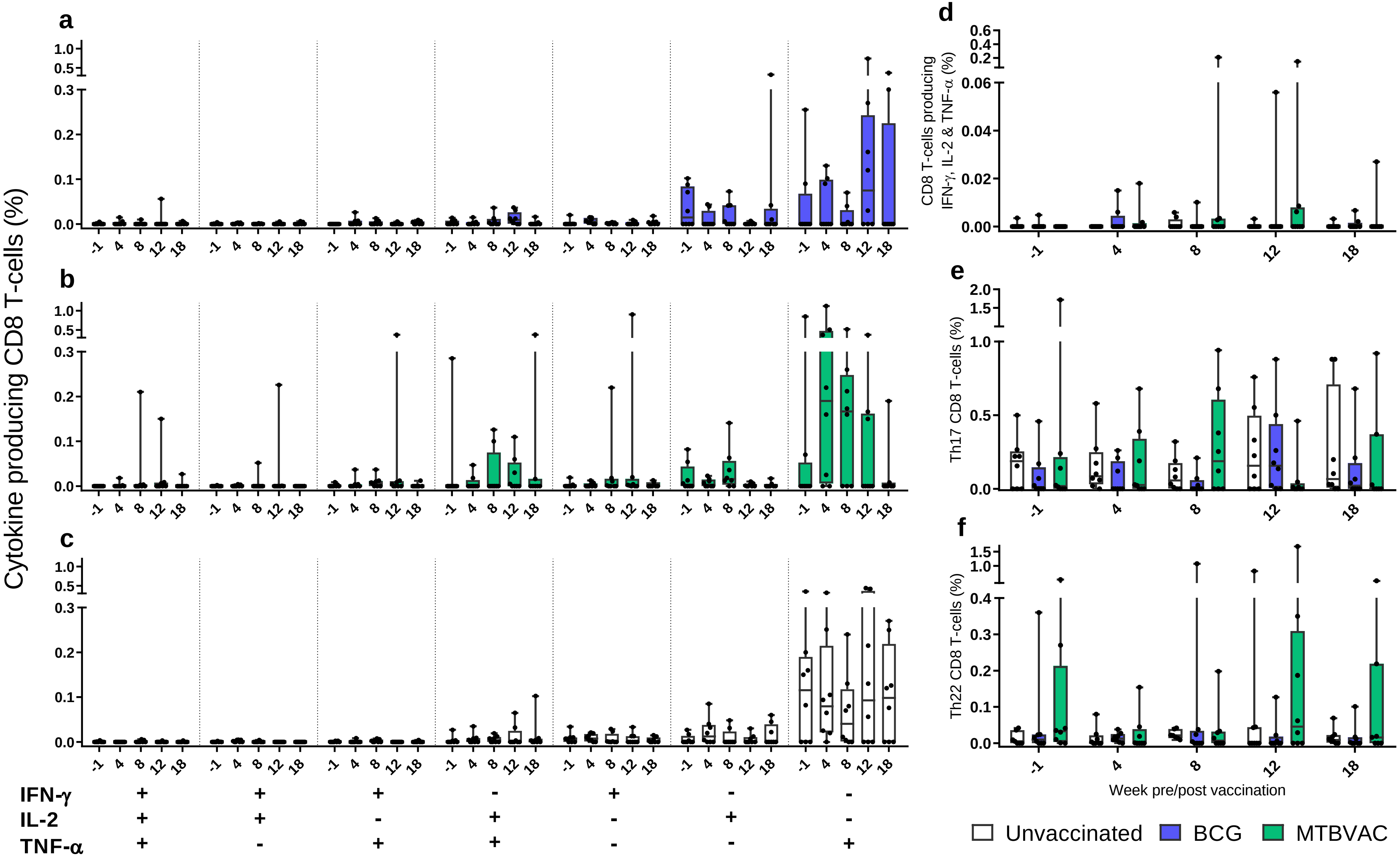

a
b
c
d
e
f
Cytokine producing CD8 T-cells (%)
IFN-γ
IL-2
TNF-α
CD8 T-cells producing IFN-γ, IL-2 & TNF-α (%)
Th17 CD8 T-cells (%)
Th22 CD8 T-cells (%)
Week pre/post vaccination
Unvaccinated
BCG
MTBVAC

Supplementary Figure 3. ***M. tuberculosis*** **peptide-specific CD8 T-cell cytokine secretion profiles measured by whole blood intracellular cytokine staining.** Box plots show the vaccination group median frequency of CD4 T-cells producing combinations of the cytokines IFN-γ, IL-2 and TNF-α (A, B, C, D); IL-17 (E) or IL-22 (F) +/- IQR with minimum and maximum values indicated by box whiskers. Cytokine production was measured at specific weeks prior to (-1) and following (4, 8, 12 and 18) BCG or MTBVAC vaccination. Frequencies measured in individual animals are represented by dots.

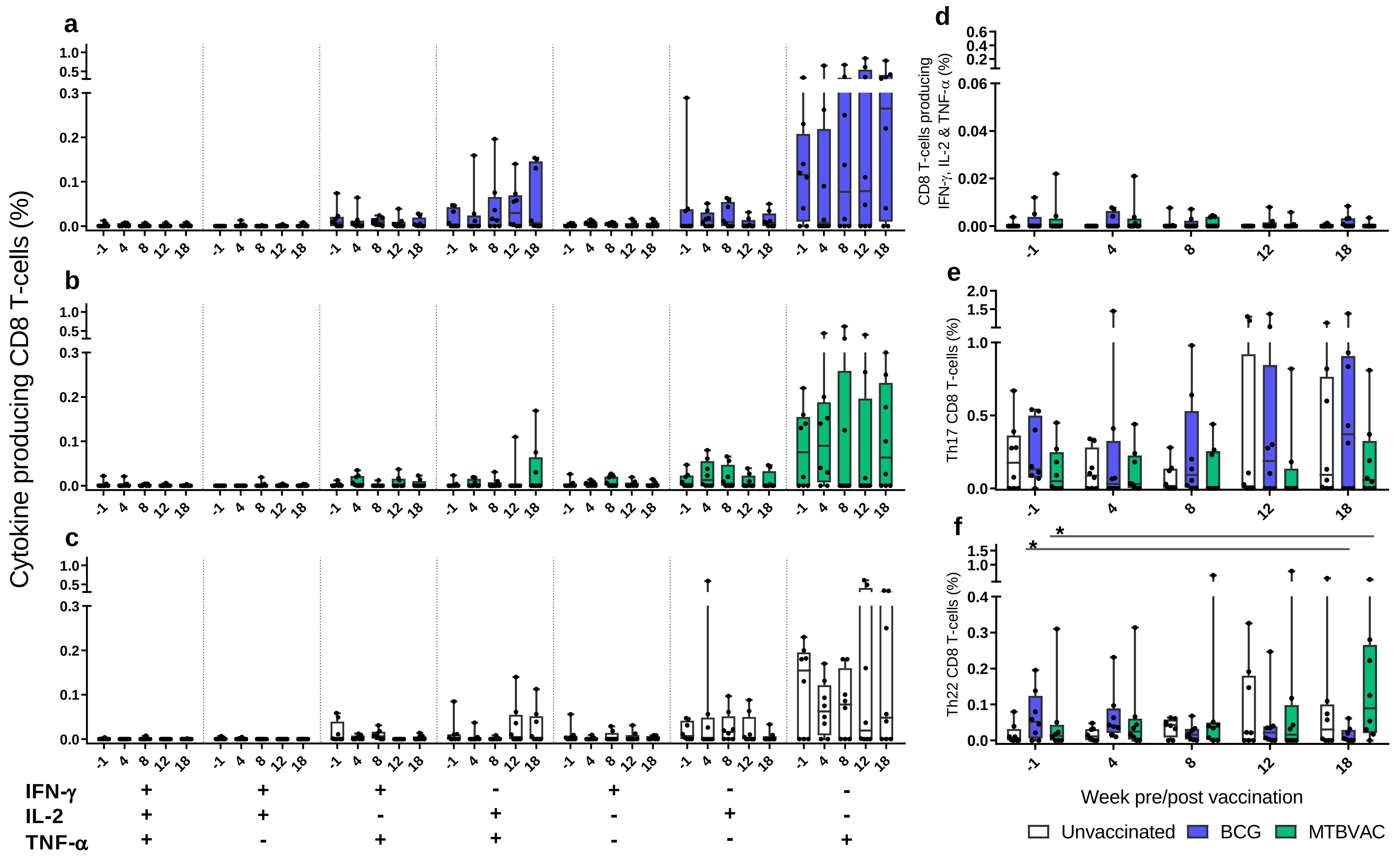

a
b
c
d
e
f
Cytokine producing CD8 T-cells (%)
CD8 T-cells producing IFN-γ, IL-2 & TNF-α (%)
Th17 CD8 T-cells (%)
Th22 CD8 T-cells (%)
IFN-γ + + + - + - -
IL-2 + + - + - + -
TNF-α + - + + - - +
Week pre/post vaccination
Unvaccinated
BCG
MTBVAC

Supplementary Figure 4. MTBVAC-specific CD8 T-cell cytokine secretion profiles measured by whole blood intracellular cytokine staining. Box plots show the vaccination group median frequency of CD4 T-cells producing combinations of the cytokines IFN-γ, IL-2 and TNF-α (A, B, C, D); IL-17 (E) or IL-22 (F) +/- IQR with minimum and maximum values indicated by box whiskers. Cytokine production was measured at specific weeks prior to (-1) and following (4, 8, 12 and 18) BCG or MTBVAC vaccination. Significant differences between pre- and post-vaccination values (Wilcoxon signed-rank) are indicated by bars and asterisks: * $p \leq 0.05$. Frequencies measured in individual animals are represented by dots.

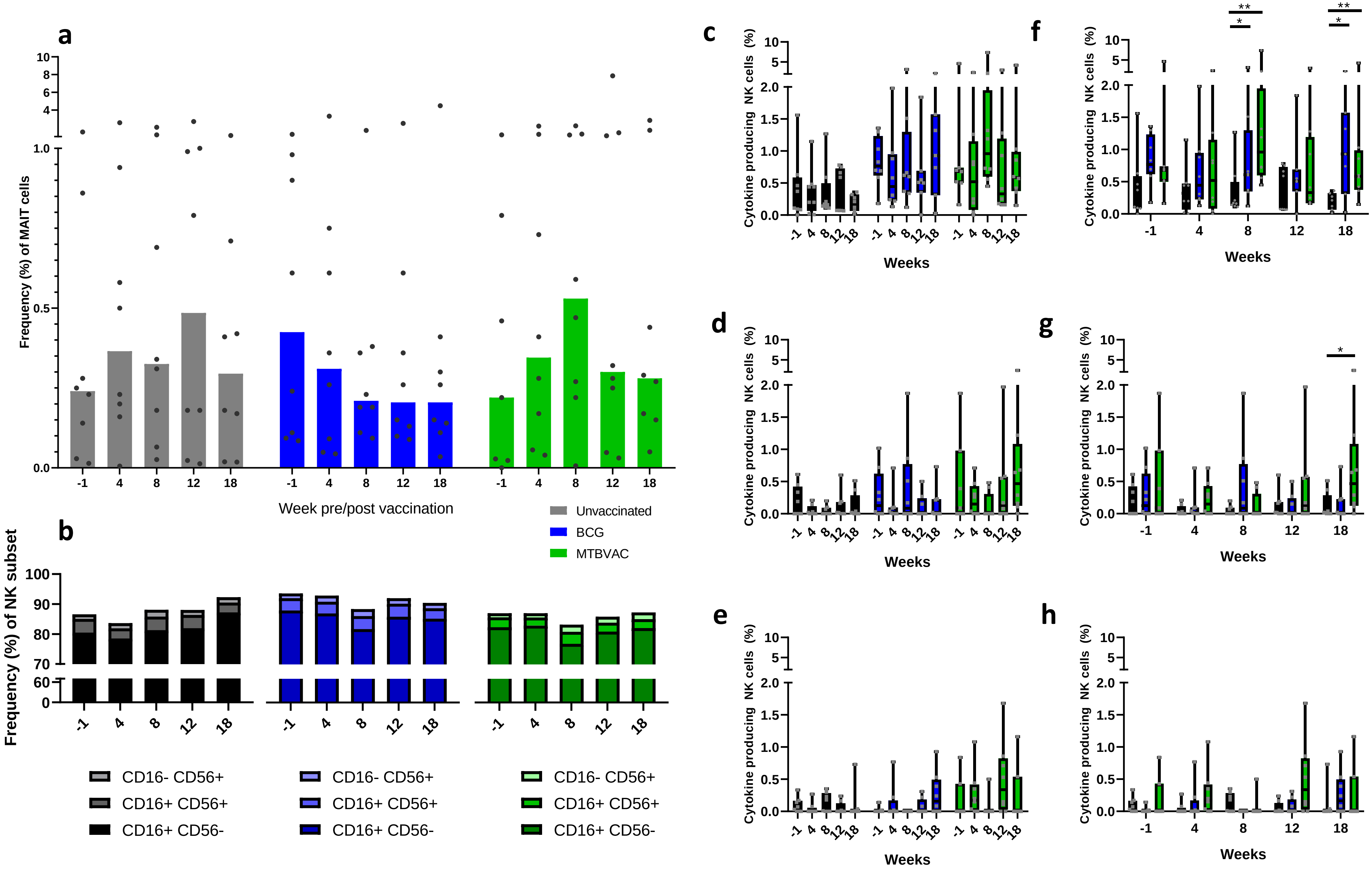

a
Frequency (%) of MAIT cells
Week pre/post vaccination
Unvaccinated
BCG
MTBVAC
b
Frequency (%) of NK subset
CD16- CD56+
CD16+ CD56+
CD16+ CD56-
c
d
e
f
g
h
Cytokine producing NK cells (%)
Weeks

Supplementary figure 5. Frequency of innate lymphoid cell populations. The frequency of mucosal associated invariant T-cells (MAIT) (A) and Natural Killer cell (B) populations were measured by WB ICS assay prior to (-1) and at weeks four, eight, 12, and 18 after BCG or MTBVAC vaccination. Figure a) bar charts show the vaccination group median percentage of MAIT cells as a proportion of the CD3+ T-cell population, frequencies measured in individual animals are shown as dots. Figure B) stacked bars show the group median percentages of each NK subset as a proportion of the CD3-, CD8+ lymphocyte population. Figures C – H) frequency of NK cells producing IFN-γ, IL-2, TNF-α, IL-17 or IL-22 in response to stimulation with *M. tuberculosis* peptides (E, H) or MTBVAC (D, G) or without antigen or peptide stimulation (C, F). Note that plots C-E show the kinetics of NK cell cytokine production in each vaccination group, whereas plots F-H show the same data aligned by time for comparative purposes. Cytokine production was measured at specific weeks prior to (-1) and following (4, 8, 12 and 18) BCG or MTBVAC vaccination. Box plots show the vaccination group median +/- IQR with minimum and maximum values indicated by box whiskers. Frequencies measured in individual animals are represented by dots. Significant differences between vaccination groups are indicated by bars with asterisks * $p \leq 0.05$, ** $p \leq 0.01$ (all p-values are unadjusted for multiple comparisons).

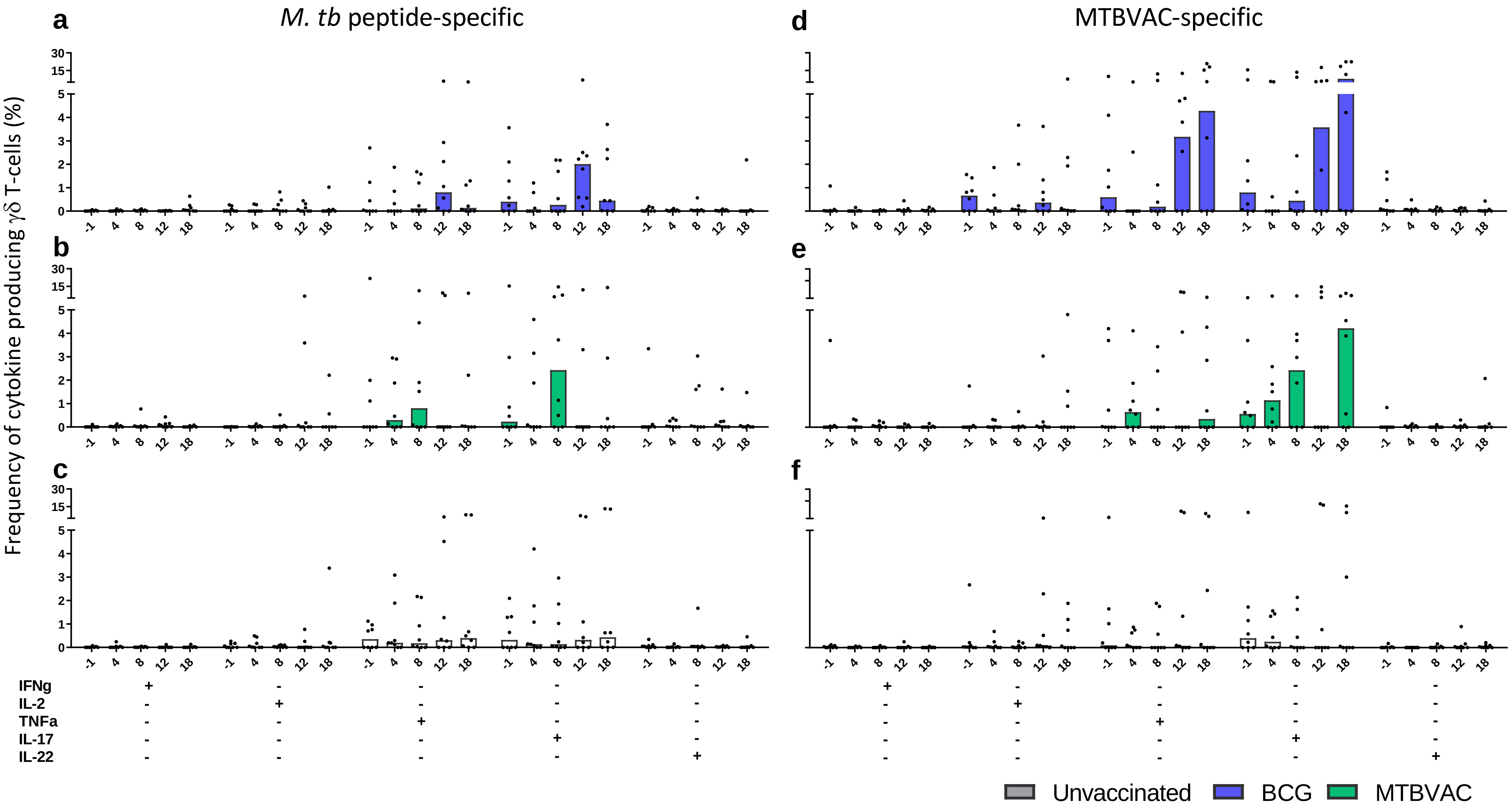

Supplementary Figure 6. Cytokine production from γδ T-cells. Bars show the vaccination group median frequency of γδ T-cells producing IFN-γ, IL-2, TNF-α, IL-17 or IL-22 in response to stimulation with *M. tuberculosis* peptides (A, B, C) or MTBVAC (D, E, F). Cytokine production was measured at specific weeks prior to (-1) and following (4, 8, 12 and 18) BCG or MTBVAC vaccination. Frequencies measured in individual animals are represented by dots. Y-axis scales are identical in all plots.

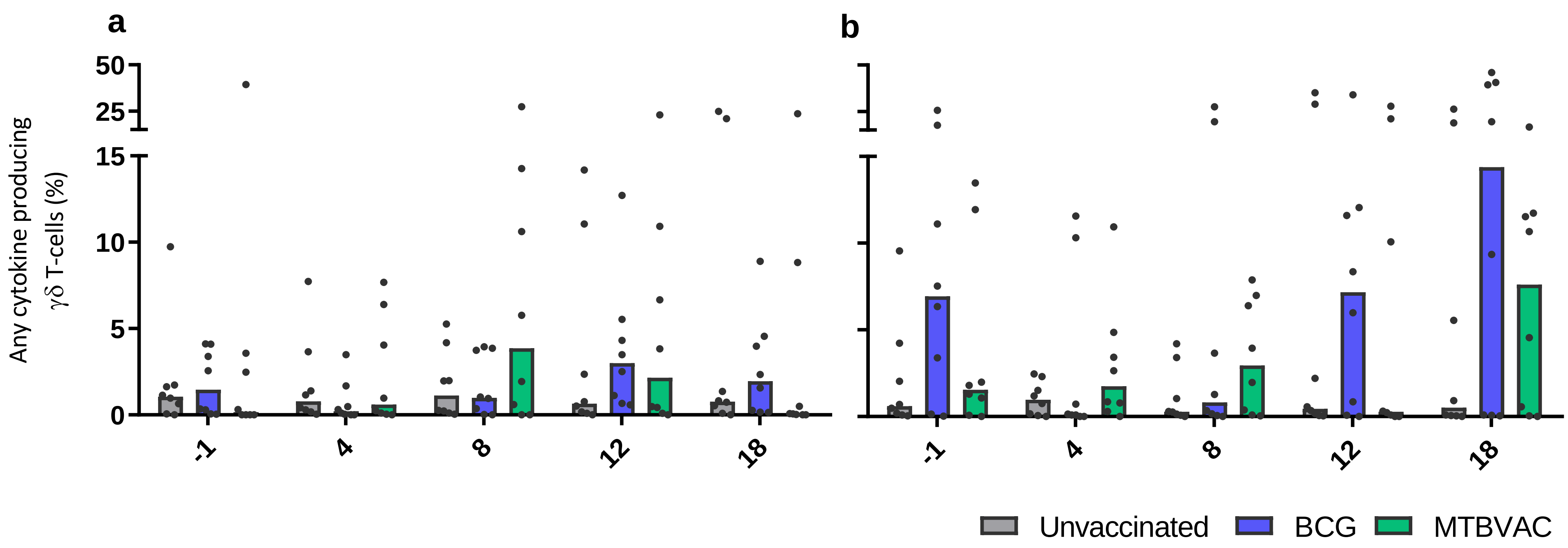

Supplementary Figure 7. Cytokine production from γδ T-cells (summed). Bars show the vaccination group median summed frequency of γδ T-cells producing IFN-γ, IL-2, TNF-α, IL-17 or IL-22 in response to stimulation with *M. tuberculosis* peptides (A) or MTBVAC (B). Cytokine production was measured at specific weeks prior to (-1) and following (4, 8, 12 and 18) BCG or MTBVAC vaccination. Frequencies measured in individual animals are represented by dots. Y-axis scales are identical in all plots.

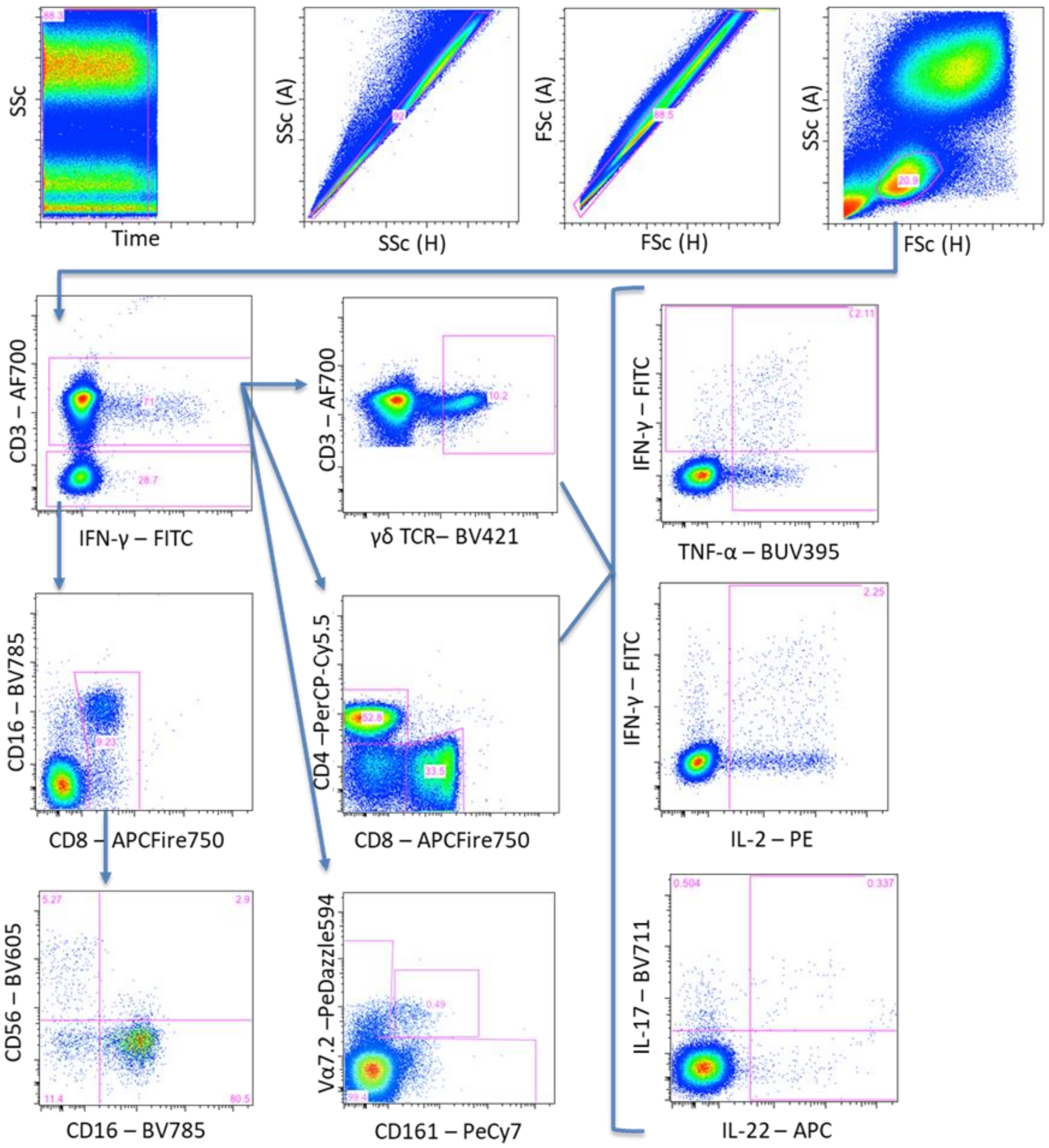

Supplementary Figure 8. Flow cytometric gating strategy. Sequential gating used for cell population analysis described in manuscript figure 4 and supplementary figures 3 – 8.
