# Supplementary material for: MTBVAC vaccination protects rhesus macaques against aerosol challenge with M. tuberculosis and induces immune signatures analogous to those observed in clinical studies
Source: NPJ Vaccines. 2021 Jan 4;6:4. doi: 10.1038/s41541-020-00262-8 (PMC7782851; doi:10.1038/s41541-020-00262-8)
Supplement: Supplementary file 2 — Reporting Summary Checklist [file 41541_2020_262_MOESM2_ESM.pdf]

nature research

Corresponding author(s): Dr Andrew White

Last updated by author(s): Nov 12, 2020

Reporting Summary

Nature Research wishes to improve the reproducibility of the work that we publish. This form provides structure for consistency and transparency in reporting. For further information on Nature Research policies, see our Editorial Policies and the Editorial Policy Checklist.

Statistics

For all statistical analyses, confirm that the following items are present in the figure legend, table legend, main text, or Methods section.

n/a	Confirmed	
☐	☒	The exact sample size (n) for each experimental group/condition, given as a discrete number and unit of measurement
☐	☒	A statement on whether measurements were taken from distinct samples or whether the same sample was measured repeatedly
☐	☒	The statistical test(s) used AND whether they are one- or two-sided<br>*Only common tests should be described solely by name; describe more complex techniques in the Methods section.*
☐	☒	A description of all covariates tested
☐	☒	A description of any assumptions or corrections, such as tests of normality and adjustment for multiple comparisons
☐	☒	A full description of the statistical parameters including central tendency (e.g. means) or other basic estimates (e.g. regression coefficient) AND variation (e.g. standard deviation) or associated estimates of uncertainty (e.g. confidence intervals)
☐	☒	For null hypothesis testing, the test statistic (e.g. F, t, r) with confidence intervals, effect sizes, degrees of freedom and P value noted<br>*Give P values as exact values whenever suitable.*
☒	☐	For Bayesian analysis, information on the choice of priors and Markov chain Monte Carlo settings
☒	☐	For hierarchical and complex designs, identification of the appropriate level for tests and full reporting of outcomes
☒	☐	Estimates of effect sizes (e.g. Cohen's d, Pearson's r), indicating how they were calculated

Our web collection on statistics for biologists contains articles on many of the points above.

Software and code

Policy information about availability of computer code

Data collection	BD FACSDiva, CTL Immunospot. Software version numbers provided in MM text.
Data analysis	FlowJo, Prism, Excel, PESTLE. Software version numbers provided in MM text.

For manuscripts utilizing custom algorithms or software that are central to the research but not yet described in published literature, software must be made available to editors and reviewers. We strongly encourage code deposition in a community repository (e.g. GitHub). See the Nature Research guidelines for submitting code & software for further information.

Data

Policy information about availability of data

All manuscripts must include a data availability statement. This statement should provide the following information, where applicable:

- Accession codes, unique identifiers, or web links for publicly available datasets
- A list of figures that have associated raw data
- A description of any restrictions on data availability

Provide your data availability statement here.

April 2020

Field-specific reporting

Please select the one below that is the best fit for your research. If you are not sure, read the appropriate sections before making your selection.

☒ Life sciences ☐ Behavioural & social sciences ☐ Ecological, evolutionary & environmental sciences

For a reference copy of the document with all sections, see nature.com/documents/nr-reporting-summary-flat.pdf

Life sciences study design

All studies must disclose on these points even when the disclosure is negative.

Sample size	See section 2.2 Vaccination, line numbers 327-330.
Data exclusions	No data excluded
Replication	Where applicable the use of replicates is reported in the MM section.
Randomization	See materials and methods section 2.1, lines 317 - 319.
Blinding	Technicians performing animal procedures and necropsies, Pathologists preparing and analysing tissue sections and radiographers reading CT scans were blinded to treatment groups.

Reporting for specific materials, systems and methods

We require information from authors about some types of materials, experimental systems and methods used in many studies. Here, indicate whether each material, system or method listed is relevant to your study. If you are not sure if a list item applies to your research, read the appropriate section before selecting a response.

Materials & experimental systems

n/a	Involved in the study	
☐	☒	Antibodies
☒	☐	Eukaryotic cell lines
☒	☐	Palaeontology and archaeology
☐	☒	Animals and other organisms
☒	☐	Human research participants
☒	☐	Clinical data
☒	☐	Dual use research of concern

Methods

n/a	Involved in the study	
☒	☐	ChIP-seq
☐	☒	Flow cytometry
☒	☐	MRI-based neuroimaging

Antibodies

Antibodies used	Antibody supplier and conjugate information is provided in the MM section.
Validation	All monoclonal antibodies were titrated to determined optimal dilutions for the assay to achieve optimal signal to noise.

Animals and other organisms

Policy information about studies involving animals; ARRIVE guidelines recommended for reporting animal research

Laboratory animals	Lines 301 - 303 of the MM section state: Twenty-four male and female rhesus macaques (Macaca mulatta) of Indian origin aged between 4.2 and 5.2 years of age were sourced from an established, characterised, closed UK breeding colony.
Wild animals	N/A
Field-collected samples	N/A
Ethics oversight	Lines 305-307 state: Animal procedures and study designs were approved by the Establishment Animal Welfare and Ethical Review Committee and authorised under a UK Home Office project licence.

Note that full information on the approval of the study protocol must also be provided in the manuscript.

Flow Cytometry

Plots

Confirm that:

☒ The axis labels state the marker and fluorochrome used (e.g. CD4-FITC).

☒ The axis scales are clearly visible. Include numbers along axes only for bottom left plot of group (a 'group' is an analysis of identical markers).

☒ All plots are contour plots with outliers or pseudocolor plots.

☒ A numerical value for number of cells or percentage (with statistics) is provided.

Methodology

Sample preparation	Full method described in MM section
Instrument	Described in the MM section
Software	Described in the MM section
Cell population abundance	N/A
Gating strategy	Gating method is described in the MM section and a full gating strategy figure is provided as supplementary data.

☒ Tick this box to confirm that a figure exemplifying the gating strategy is provided in the Supplementary Information.
